# Development and external validation of a prediction model for patient‐relevant outcomes in patients with chronic widespread pain and fibromyalgia

**DOI:** 10.1002/ejp.1937

**Published:** 2022-03-15

**Authors:** V. P. Moen, A. T. Tveter, R. D. Herbert, K. B. Hagen

**Affiliations:** ^1^ Centre for Habilitation and Rehabilitation Haukeland University Hospital Bergen Norway; ^2^ Department of Health and Functioning Western Norway University of Applied Sciences Bergen Norway; ^3^ National Advisory Unit on Rehabilitation in Rheumatology Department of Rheumatology Diakonhjemmet Hospital Oslo Norway; ^4^ Department of Physiotherapy OsloMetropolitan University Oslo Norway; ^5^ Neuroscience Research Australia (NeuRA) Sydney Australia; ^6^ School of Medical Sciences University of New South Wales Sydney Australia; ^7^ Division of Health Services Norwegian Institute of Public Health Oslo Norway

## Abstract

**Background:**

The objective of this study was to develop prediction models and explore the external validity of the models in a large sample of patients with chronic widespread pain (CWP) and fibromyalgia (FM).

**Methods:**

Patients with CWP and FM referred to rehabilitation services in Norway (*n* = 986) self‐reported data on potential predictors prior to entering rehabilitation, and self‐reported outcomes at one‐year follow‐up. Logistic regression models of improvement, worsening and work status, and a linear regression model of health‐related quality of life (HRQoL), were developed using lasso regression. Externally validated estimates of model performance were obtained from the validation set.

**Results:**

The number of participants in the development and the validation sets was 771 and 215 respectively; only participants with outcome data (*n* = 519–532 and 185, respectively) were included in the analyses. On average, HRQoL and work status changed little over one year. The prediction models included 10–11 predictors. Discrimination (AUC statistic) for prediction of outcome at follow‐up was 0.71 for improvement, 0.67 for worsening, and 0.87 for working. The median absolute error of predictions of HRQoL was 0.36 (0.22–0.51). Reasonably good predictions of working at follow‐up and HRQoL could be obtained using only the baseline scores as predictors.

**Conclusions:**

Moderately complex prediction models (10–11 predictors) generated poor to excellent predictions of patient‐relevant outcomes. Simple prediction models of working and HRQoL at follow‐up may be nearly as accurate and more practical.

**Significance:**

Prediction modelling of outcome in rehabilitation has been sparsely explored. Such models may guide clinical decision‐making. This study developed and externally validated prediction models for outcomes of people with chronic widespread pain and fibromyalgia in a rehabilitation setting. Multivariable prediction models generated poor to excellent predictions of patient‐relevant outcomes, but the complexity of these models may reduce their clinical utility. Simple univariable prediction models were nearly as accurate and may have more potential for use in clinical practice.

## 1 INTRODUCTION

Chronic widespread pain (CWP) and fibromyalgia (FM) pose major societal challenges in terms of prevalence (Kinge et al., 2015), non‐fatal health loss (Knudsen et al., 2017) and costs (Folkehelseinstituttet, 2015). Chronic widespread pain and FM are interrelated illnesses in which pain is the dominant symptom and other symptoms such as fatigue, non‐refreshing sleep, depression and cognitive impairment are common but not universal (Wolfe et al., 2016). The symptoms may result in reduced quality of life, impaired physical functioning including reduced work ability, and increased sick absence, and may initiate extensive use of medical care (Turk et al., 2008).

There is no curative treatment for CWP and FM. The effects of pharmacological treatments are of questionable clinical relevance, and there is little evidence of the effectiveness of non‐pharmacological treatments (Nuesch et al., 2013). In Norway, patients with CWP and FM often undergo rehabilitation consisting of interdisciplinary interventions addressing both cognitive and functional aspects of the health status of the patient. Since the pathogeneses of CWP and FM remain unclear, the therapeutic focus is often on cognitive and behavioural components of pain and disability (de Rooij et al., 2013) as well as symptom reduction (Nuesch et al., 2013).

For patients, it is important to know whether the intervention they are undergoing is safe and has a beneficial effect. At the same time, patients also wish to know their prognosis, both at the time of diagnosis and when entering a rehabilitation program. The prognosis of CWP and FM has been little explored in specialist rehabilitation services in Norway. A better understanding of prognosis could provide valuable decision support. The substantial level of heterogeneity within individuals with CWP and FM (de Rooij, van der Leeden, et al., 2013) suggests that a stratified management approach might lead to more specific and better management of these patients.

Systematic reviews have summarized the evidence across a range of musculoskeletal conditions and found moderate to strong evidence that widespread pain, high functional disability, somatization, intense pain, long pain duration and high depression/anxiety scores are generic predictors for poor prognosis (Artus et al., 2017; Tseli et al., 2019). Additionally, domain‐specific measures, such as self‐efficacy beliefs, are correlated with key outcomes in chronic pain populations (Jackson et al., 2014). While improvements are often measured with self‐reported physical and cognitive dimensions of health (Tseli et al., 2019), the post‐rehabilitation working status of these patients has been explored to a lesser extent.

There is a need to develop clinical prediction tools for health outcomes for patients with musculoskeletal conditions (Tseli et al., 2019). To our knowledge, no studies have developed a prediction model and explored the external validity of the model in a large sample of patients with CWP and FM. Hence, the objective of this study was to develop models to predict health outcomes at one year in patients with CWP and FM presenting to specialized rehabilitation centres, and to test the models’ performance, including their external validity.

## 2 METHOD

### 2.1 Study design and participants

The study is based on a cohort recruited from patients admitted to specialized rehabilitation centres in Norway between March 2017 and December 2018. Adults between 18 and 70 years old with CWP or FM as the main diagnosis were invited to participate (study‐eligible *n* = 3089). Patients with insufficient Norwegian language skills to complete questionnaires and patients with other chronic diagnoses as their main diagnoses were excluded. Patients received a postal information letter, and written informed consent was obtained from all participants. Procedures conformed to the Helsinki Declaration of 1975, as revised in 1983, and the protocol was approved by the Regional Ethics Committee South East in Norway (REK‐No. 2016/2032). Patient‐reported data were collected either electronically (www.infopad.no) or on paper, according to the patient's preferences. All patient‐reported data were provided by the individual from home, and data at baseline were collected between two and four weeks prior to admittance to a rehabilitation centre. Follow‐up was six months and one year after baseline. Two reminders (both electronic and paper) were given. The study was conducted in accordance with the TRIPOD statement.

### 2.2 Potential prognostic variables

Activity impairment was assessed using the Work Productivity and Activity Impairment (WPAI) questionnaire. The WPAI assesses work ability, including work missed, impairment while working, overall work impairment, and activity impairment. Only the subscore regarding activity impairment during the previous seven days was used in this project, calculated and presented as a percentage score. The instrument has shown adequate reproducibility in employed individuals affected by a health problem (Reilly et al., 1993), and the instrument reports valid scores for assessing impairments in paid work and activities in patients with rheumatoid arthritis (Zhang et al., 2010).

Pain and psychological distress was measured with the long form of Örebro Musculoskeletal Pain Questionnaire (ÖMPQ). The ÖMPQ comprises 21 items concerning pain and psychological distress. It is designed to identify people with musculoskeletal pain and distress who are at risk of developing prolonged symptoms. The scores range from 0 to 210, with higher scores representing more pain and distress. The instrument has sound psychometric properties in populations with neck and back disorders (Hilfiker et al., 2016; Langenfeld et al., 2018), and in patients with low back pain it has acceptable and reasonable predictive validity for disability outcomes and persistent pain respectively, and excellent predictive validity for absenteeism outcomes (Dagfinrud et al., 2013; Karran et al., 2017; Maher & Grotle, 2009).

Pain intensity was measured on a scale from 0 to 10, using item 10 of the ÖMPQ, with higher scores representing more intense pain. Pain duration was measured as the number of years with pain.

Persistent disabling symptoms was assessed with the Keele STarT Back Screening Tool. It comprises 8 statements which the patients are asked to agree or disagree with, and one item in which the patients score the bothersomeness of his/her back pain on a 5‐point Likert scale anchored at “Not at all” and “Extremely,” Four items explicitly concern the last two weeks. For the use in this population, the instrument was modified by deletion of the first question on sciatic pain and the rephrasing of “back pain” to “musculoskeletal pain.” The overall score ranged from 0 to 8, with higher scores representing more distress. In addition to high reliability, the instrument has been shown to predict health‐related quality of life, work ability, global improvement, pain severity, disability, catastrophizing and fear in populations with neck and back pain (Forsbrand et al., 2018; Robinson & Dagfinrud, 2017; Wideman et al., 2012). Furthermore, the instrument has been used to demonstrate both clinical effect and cost‐effectiveness of a stratified management approach (Hill et al., 2011).

The severity of fibromyalgia was measured with the Fibromyalgia Poly‐symptomatic Distress Scale. The instrument consists of two separate sub‐scales: the Widespread Pain Index which assesses the number of areas in which the patient has had pain over the last week (score ranging from 0 to 19), and the Symptom Severity Scale which assesses fatigue, quality of sleep and cognitive symptoms (score ranging from 0 to 12, with higher scores for greater severity). The instrument is a valid tool for the assessment of fibromyalgia and can validly differentiate severity subgroups with FM (Fors et al., 2020; Wolfe et al., 2015).

The Activity Index is based on three items: frequency, intensity and duration of exercise during the past week. The calculated scores range from 0 to 15 with higher scores indicating higher activity levels (Kurtze et al., 2008). The index which has been used in a large survey in Norway (HUNT‐study) provides a useful measure of leisure‐time physical activity and is an appropriate tool for use in epidemiological studies (Kurtze et al., 2008).

Self‐efficacy was measured by the subscales for pain and symptoms from the Arthritis Self‐Efficacy Scale (ASES), scored on a 5‐point Likert scale from “very uncertain” to “very certain.” The scores range from 0 to 20 for the pain subscale and 0 to 24 for the symptom subscale with higher score representing higher levels of self‐efficacy. The instrument has been tested for validity and reliability (Garratt et al., 2017; Wilcox et al., 2014).

Anxiety and depression were measured with single‐item screening questions taken from the Subjective Health Complaints Inventory (Eriksen et al., 1999), with minor modifications by Reme et al (Reme et al., 2014). The questions assess whether, and to what extent, patients have been affected in the last 30 days, ranging from 0 to 3 (0 = not at all, 3 = serious). The single‐item questions have good/excellent performance in detecting depression and fair/good performance in detecting anxiety disorders in patients with low back pain (Reme et al., 2014).

Comorbidity was defined as two or more coinciding diagnoses/disorders/health conditions in the same individual (Mercer et al., 2009). Eighteen items were included, with 17 specified specific diagnoses/disorders and one item enabled participants to specify other diagnoses/disorders/health conditions themselves.

Health‐related quality of life at baseline was measured with the EuroQol EQ‐5D‐5L. The EQ‐5D‐5L consists of 5 questions concerning functional level, pain and psychological distress, and a visual analogue scale in which the patients rate their health status (0–100, with higher scores indicating better health). A utility score (values ≤ 1, with higher scores for better health) was calculated based on the five questions. This instrument is widely used, and its measurement properties have been well documented (Janssen et al., 2013).

Working status at baseline was provided from the WPAI questionnaire where the patients answered if they were working or not working.

### 2.3 Outcome variables

The primary outcome was global improvement after rehabilitation measured with the Patient Global Impression of Change (PGIC) scale (six months and) one year after baseline. PGIC is a 7‐point self‐reported Likert scale ranging from 1 (“I feel very much worse”) through 4 (“no change”) to 7 (“I feel very much better”). The scale was dichotomized by collapsing scores of 1–5 (not improved) and 6–7 (improved). Scores of 6 and 7 are considered to represent clinically relevant improvement (Choy et al., 2009).

Secondary outcomes were global worsening, working status and health‐related quality of life (six months and) one year after baseline. Worsening was defined by collapsing PGIC scores of 1–2 (worsening) and 3–7 (not worse). Working status was dichotomized into working or not working at follow‐up. Health‐related quality of life was measured with the EQ‐5D‐5L.

### 2.4 Sample size

A sample size of 600 participants was expected to include at least 100 participants who experienced improvement. As there were 10 putative predictors, this yields 10 “events” per predictor (Peduzzi et al., 1996). To allow for a 15% non‐response at one year follow up, we sought to recruit 700 participants into the development arm of the cohort.

Norway is divided into four health regions, three of which were included in the present study. Prior to the conduct of the study, a decision was made to divide the cohort into two parts: participants from the South‐Eastern and the Middle health regions were included in the development set, and participants from the Western health region were included in the validation set.

### 2.5 Statistical analysis

Descriptive statistics were used to summarize baseline characteristics of enrolled participants in both the development set and validation set. Completeness of data is also reported.

The 1‐year follow‐up was the primary endpoint. However, some participants (124 (16%) in the development set and 24 (11%) in the validation set) responded only to the 6‐month follow‐up (not to the 12‐month follow‐up). These participants’ outcomes were carried forward to 12 months. We refer to these data, consisting of 12‐month follow‐up data for most participants and, for some participants, 6‐month data carried forward, as the follow‐up data.

Differences in outcomes for patients answering both at 6 and 12 months were analysed with chi‐square for categorical variables and paired *t*‐test for EQ‐5D‐5L.

Only participants with outcome data were included in the analyses. The development set was used to build a predictive model. For the primary analysis, a logistic regression model was used to predict the primary outcome, i.e., improvement. For analysis of secondary outcomes, logistic models were used to predict worsening and working status, and a linear regression model was used to predict health‐related quality of life. The predictors were baseline health‐related quality of life, WPAI impairment working, WPAI Impairment activity, pain intensity, pain duration, arthritis self‐efficacy scale pain, arthritis self‐efficacy scale symptoms, ÖMPQ score, comorbidities, anxiety, depression, widespread pain index, symptom severity, and the StartBack screening tool score. Lasso regression was used to generate parsimonious models (i.e., to select predictor variables) and to shrink the regression coefficients. The value for lambda, which penalizes model complexity, was determined using cross‐validation. Penalized regression coefficients are presented.

Once the prediction models (i.e., the selected variables and their penalized regression coefficients) had been identified, the models were fixed and model performance (discrimination and calibration) was assessed by averaging performance in 1000 bootstrap replications of the development set (internal validation) and in the validation set (external validation).

The discrimination of models for the three binary outcomes (improvement, worsening, working status) was examined by inspecting plots of the distributions of predicted probabilities amongst participants who did and did not experience the outcome of interest, and with receiver operating characteristic (ROC) curves. The area under the ROC curve (the AUC statistic) was used to quantify discrimination. For the development sample, optimism of the AUC was assessed in bootstrap samples using the procedure described by Steyerberg (Steyerberg, 2009). Optimism‐corrected AUCs are reported. AUCs were interpreted as follows: <0.6 = non‐informative, 0.6 to 0.7 = poor discrimination, 0.7 to 0.8 = acceptable discrimination, 0.8 to 0.9 = excellent discrimination, and >0.9 = outstanding discrimination, consistent with previous studies (Beneciuk et al., 2018; Karran et al., 2017; Traeger et al., 2015).

The performance of predictions of health‐related quality of life was quantified with the R^2^ statistic (proportion of variance explained by the prediction model) and the median absolute error of the predictions.

Additional linear and logistic regression models were constructed with the EQ‐5D‐5L and working status at follow‐up as outcomes. In these models, the baseline value (EQ‐5D‐5L or working status) was included as the only predictor.

The calibration of the models was examined in two ways. First, “calibration in the large” was quantified by comparing mean observed and predicted outcomes. Second, plots of predicted versus observed outcomes were inspected. The calibrationbelt procedure described by Nattino and colleagues (Nattino et al., 2016) was used to formally test goodness of fit of the three models with binary outcomes. ROC curves and calibration plots are presented.

## 3 RESULTS

**FIGURE 1:**
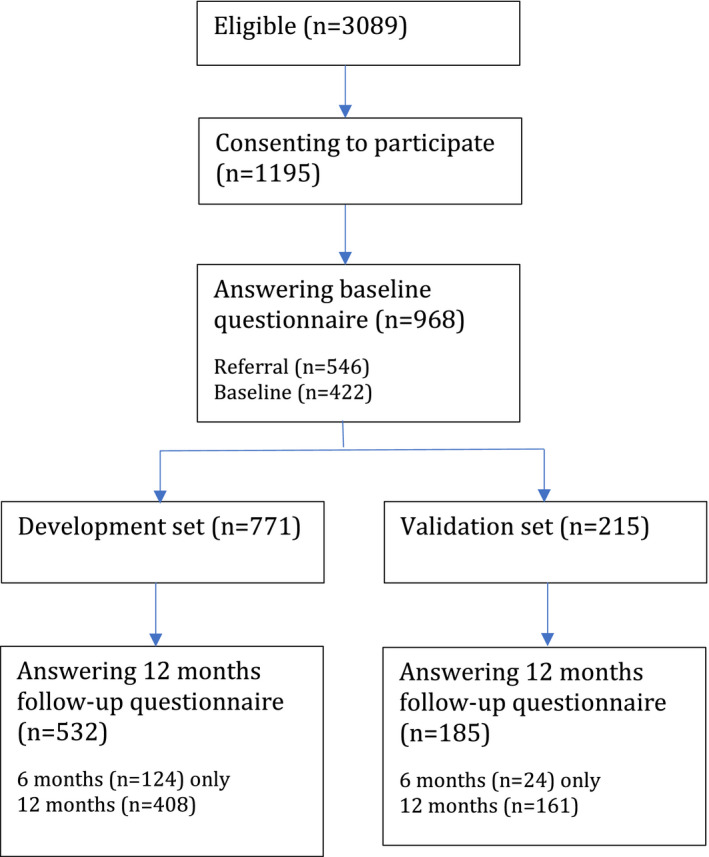
Flow of people through the study

The number of the participants was 986, of whom 771 were in the development set and 215 were in the validation set. The baseline characteristics of participants in the development and validation sets are shown in Table 1. The completeness of the data is reported in Table 2. The models were developed on the subset of participants who provided outcome data. The flow of participants through the study is shown in Figure 1. We have no data from non‐participants.

**TABLE 1:** Baseline characteristics of participants in development and validation sets

	Development set (n = 737–771)	Validation set (n = 205–210)
Age (years)^a^	49.0 (41.0 to 54.0)	49.0 (41.0 to 56.0)
Gender male: female^b^	63 (8.4%): 686 (91.6%)	22 (10.7%): 183 (89.3%)
BMI^a^	27.7 (24.2 to 32.2)	28.2 (24.3 to 32.2)
Education <12 years: ≥12 years^b^	479 (63.9%): 371 (36.1%)	131 (63.3%): 76 (36.7%)
Working: not working^b^	291 (39.0%): 456 (61.0%)	90 (43.3%): 118 (56.7%)
Living together: living alone^b^	506 (66.9%): 250 (33.1%)	151 (72.2%): 58 (27.8%)
Disease duration (years)^a^	10.0 (5.0 to 20.0)	14.0 (6.0 to 20.0)

^a^ Medians (first and third quartiles).

^b^ Counts (column percentages).

**TABLE 2:** Completeness of data

	Development set (*N* = 771)		Validation set (*N* = 215)	
	Baseline	12 months	Baseline	12 months
**Outcomes**				
Primary				
Global improvement		519 (67.3%)		185 (86.0%)
Secondary				
Global worsening		519 (67.3%)		185 (86.0%)
Working status		526 (68.2%)		185 (86.0%)
Health‐related quality of life		532 (69.0%)		185 (86.0%)
Predictors				
Health‐related quality of life	757 (98.2%)		209 (97.2%)	
WPAI Impairment working	747 (96.9%)		208 (96.7%)	
Örebro musculoskeletal screening tool	757 (98.2%)		209 (97.2%)	
STarT Back screening tool	756 (98.1%)		209 (97.2%)	
WPAI Impairment activity	747 (96.9%)		208 (96.7%)	
Widespread pain index	756 (98.1%)		210 (97.7%)	
Activity index	752 (97.5%)		207 (96.2%)	
Symptom severity scale	756 (98.1%)		210 (97.7%)	
Arthritis self‐efficacy scale pain	757 (98.2%)		209 (97.2%)	
Arthritis self‐efficacy scale symptoms	757 (98.2%)		209 (97.2%)	
Anxiety	754 (97.8%)		209 (97.2%)	
Depression	753 (97.7%)		210 (97.7%)	
Pain intensity	757 (98.2%)		209 (97.2%)	
Pain duration	741 (96.1%)		205 (95.3%)	
Comorbidities	758 (98.3%)		210 (97.7%)	

^a^ Data are n (% of N).

For patients who provided both 6‐ and 12‐month outcomes, there were no significant differences between the outcomes at the two time points for any of the primary or secondary outcomes (*p* > 0.05).

The length of the rehabilitation stay and the proportion of participants who received inpatient or outpatient rehabilitation are reported in Table 3.

**TABLE 3:** Type and length of the rehabilitation stay among participants

	Development set (*n* = 497 (64.5%))	Validation set (*n* = 179 (83.3%))
Inpatient: Outpatient^a^	414 (83.3%): 83 (16.7%)	149 (83.2%): 30 (16.8%)
Length of stay, weeks^a^	4 (3 to 5)	4 (3 to 4)

^a^ Counts (column percentages).

^b^ Medians (first and third quartiles).

In the development set, 58 participants (11.2%) improved and 85 (16.4%) worsened. In the validation set, 15 participants (8.1%) improved and 24 (13.0%) worsened. For the development and validation sets respectively, 291 (39.0%) and 90 (43.3%) were working at baseline, and 175 (33.3) and 69 (37.3%) were working at follow‐up. The mean (SD) EQ‐5D‐5L index at baseline was 0.45 (0.24) and 0.51 (0.23) at baseline, and 0.45 (0.25) and 0.48 (0.22) at follow‐up, for the development and validation sets respectively.

The optimization procedures retained between 10 and 11 variables with non‐zero regression coefficients in the prediction models (Table 4). The prediction model for improvement had just acceptable discrimination (AUC in the validation set of 0.71; Table 5) and the prediction model for worsening had poor discrimination (AUCs of 0.67; Table 5). The model predicting working at follow‐up had excellent discrimination (AUC 0.87; Table 5). The R^2^ (and median absolute error) of the model predicting health‐related quality of life was 0.38 (0.31–0.45) for the optimism‐corrected model in the development set and 0.36 (0.22–0.51) for the validation set. All four models were well calibrated (data not shown).

**TABLE 4:** Final regression models (penalized coefficients) for the primary and secondary outcomes

	Coefficient
**Primary outcome**	
Global improvement	
Intercept	−0.804
Health‐related quality of life	0.368
WPAI Impairment working	−0.007
Pain duration	−0.003
Arthritis self‐efficacy scale pain	0.071
Arthritis self‐efficacy scale symptoms	−0.048
StartBack screening tool	−0.108
Örebro musculoskeletal screening tool	−0.005
Comorbidities	−0.063
WPAI Impairment activity	−0.006
Widespread pain index	0.041
Symptom severity scale	−0.009
**Secondary outcomes**	
Global worsening	
Intercept	−5.309
Health‐related quality of life	−0.853
Pain intensity	0.104
Pain duration	−0.025
Arthritis self‐efficacy scale pain	−0.040
Arthritis self‐efficacy scale symptoms	0.100
Örebro musculoskeletal screening tool	0.012
Comorbidities	0.143
Anxiety	0.054
WPAI Impairment activity	0.013
Widespread pain index	0.006
Working status	
Intercept	4.236
WPAI Impairment working	−0.040
Pain intensity	−0.225
Pain duration	−0.018
Arthritis self‐efficacy scale pain	0.024
Arthritis self‐efficacy scale symptoms	−0.024
StartBack screening tool	0.063
Örebro musculoskeletal screening tool	−0.038
Anxiety	0.057
WPAI impairment activity	0.009
Widespread pain index	0.007
Symptom severity scale	0.074
Health‐related quality of life	
Intercept	0.716
Health‐related quality of life	0.304
WPAI Impairment working	−0.00005
Pain intensity	−0.003
Arthritis self‐efficacy scale symptoms	−0.001
StartBack screening tool	−0.012
Activity index	0.004
Comorbidities	−0.006
Anxiety	−0.009
WPAI Impairment activity	−0.001
Widespread pain index	−0.001
Symptom severity scale	−0.010

**TABLE 5:** Discriminative performance (AUC) of the multivariable predictive models.

	Optimism‐adjusted	External
Primary outcome		
Global improvement	0.73 (0.66–0.80)	0.71 (0.58–0.85)
Secondary outcomes		
Global worsening	0.75 (0.70–0.81)	0.67 (0.56–0.79)
Working status	0.87 (0.83–0.90)	0.87 (0.82–0.93)

Nearly as good predictions of EQ‐5D‐5L at follow up could be obtained using EQ‐5D‐5L at baseline as the only predictor: The regression model was EQ‐5D‐5L at follow up = 0.18 + 0.58 × EQ‐5D‐5L at baseline in the development set and 0.20 + 0.55 × EQ‐5D‐5L in the validation set. The adjusted r^2^ of this model was 0.30 in the development set and 0.34 in the validation set. Likewise, nearly as good predictions of working at follow‐up could be obtained using working at baseline as the only predictor: *The model was log odds of working at follow*‐*up* = *0*.*10* × *exp(3*.*11* × *working at baseline) (i*.*e*., *OR* = *22*.*3) for the development set*, *and 0*.*09* × *exp(3*.*58* × *working at baseline) (i*.*e*., *OR* = *35*.*7) for the validation set*. *In these equations*, *not working at baseline is assigned a value of 0 and working at baseline is assigned a value of 1*.

**FIGURE 2:**
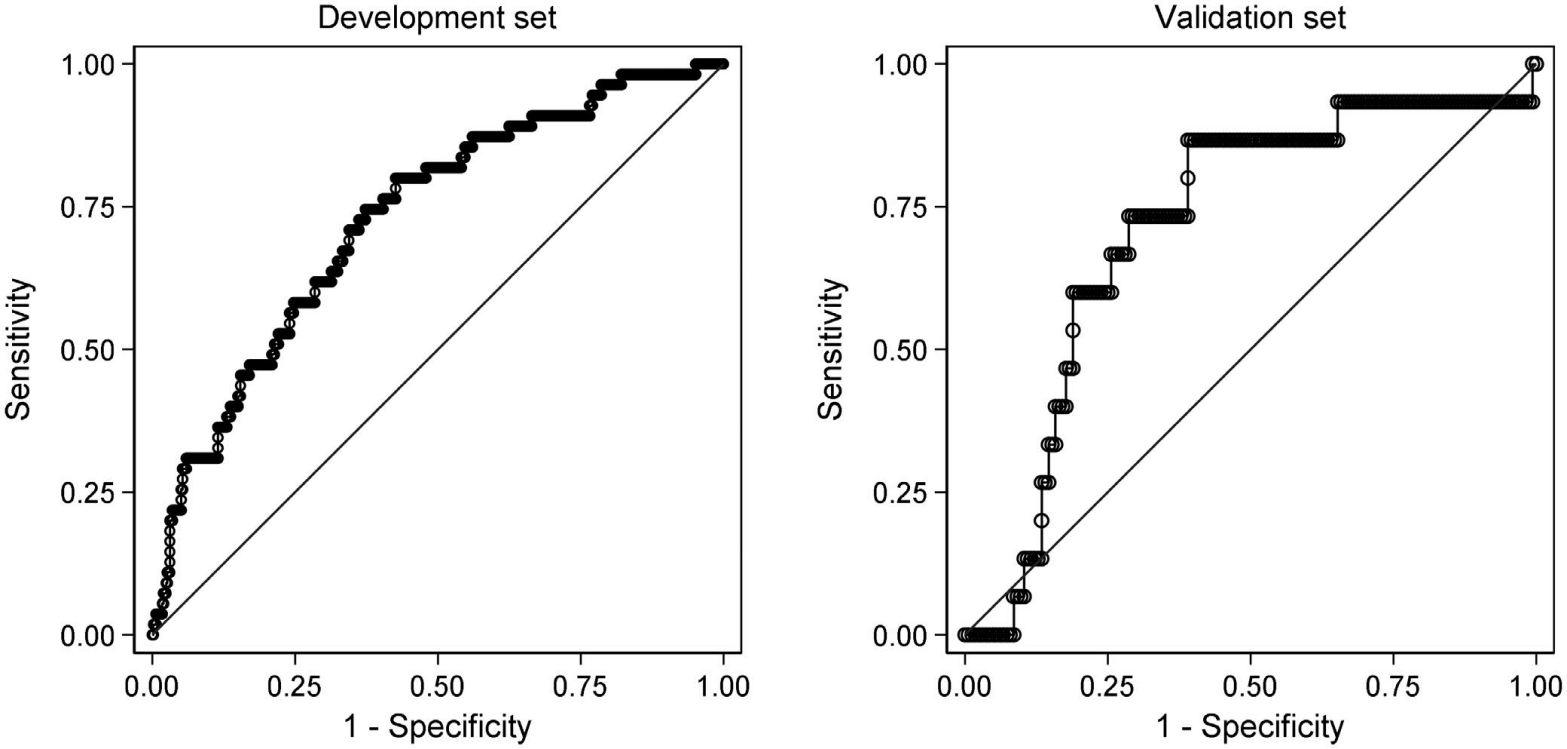
ROC curves of the multivariable model for global improvement

**FIGURE 3:**
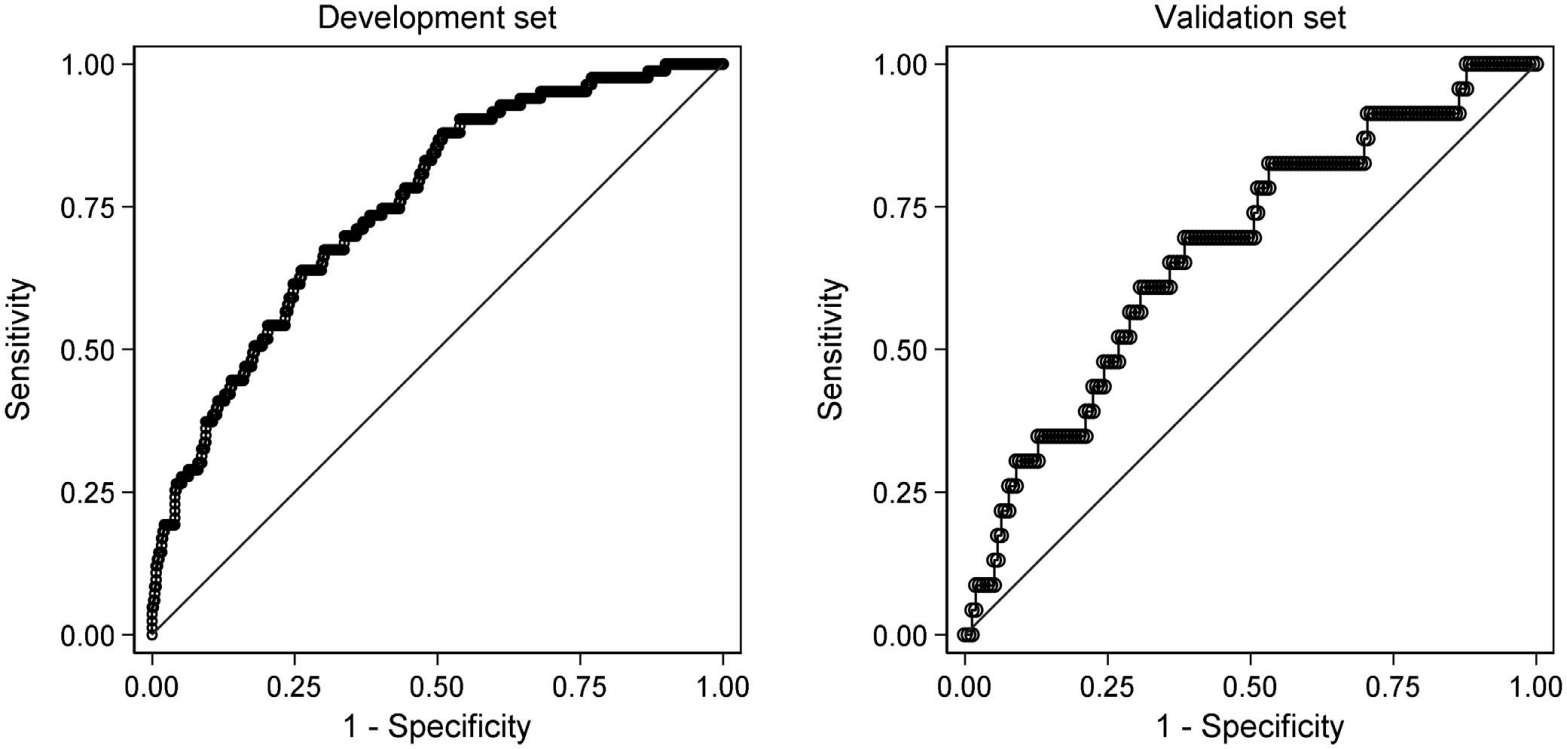
ROC curves of the multivariable model for global worsening

**FIGURE 4:**
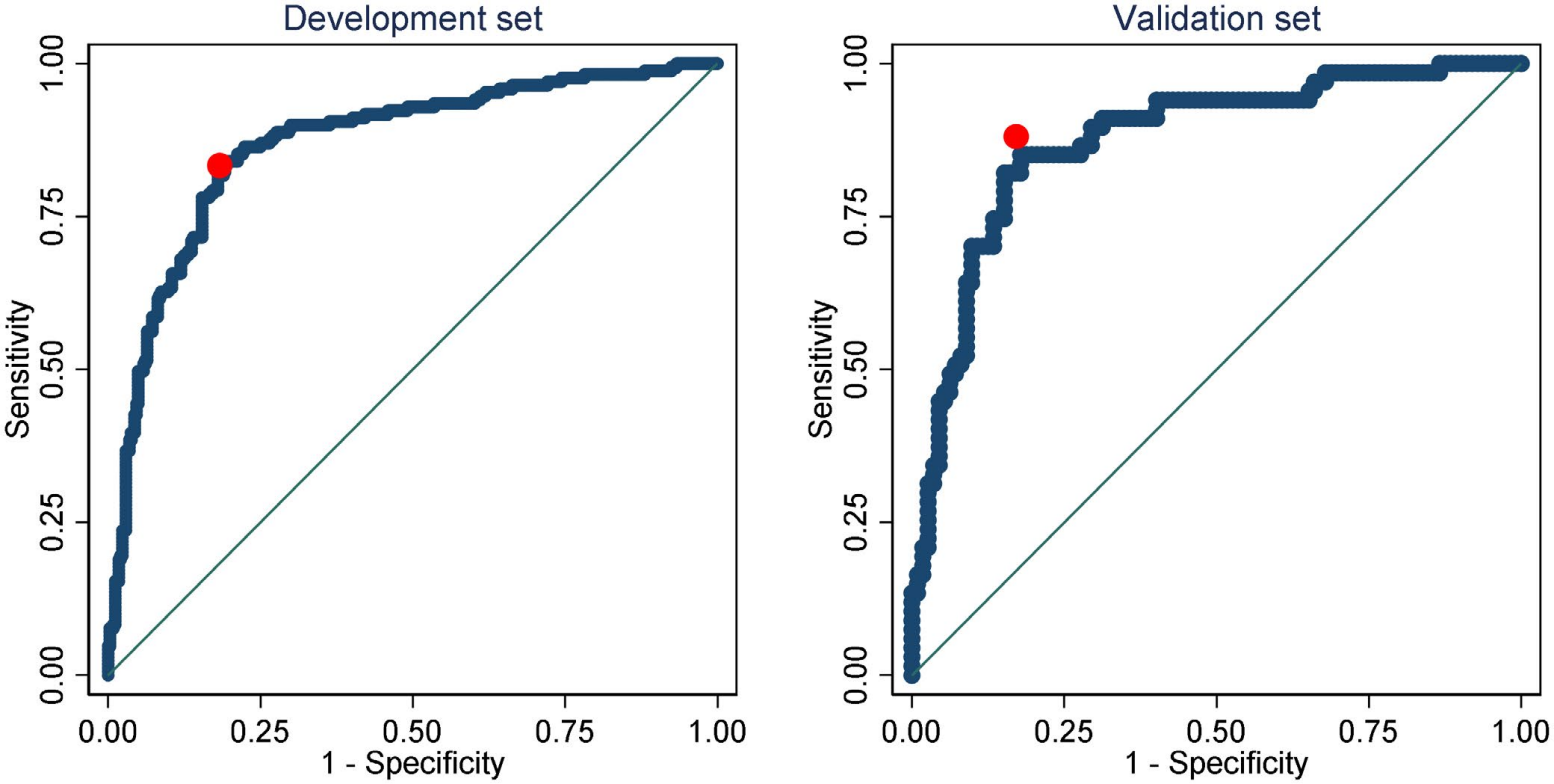
ROC curves of the multivariable (black) and the simple model (red) for working status

**FIGURE 5:**
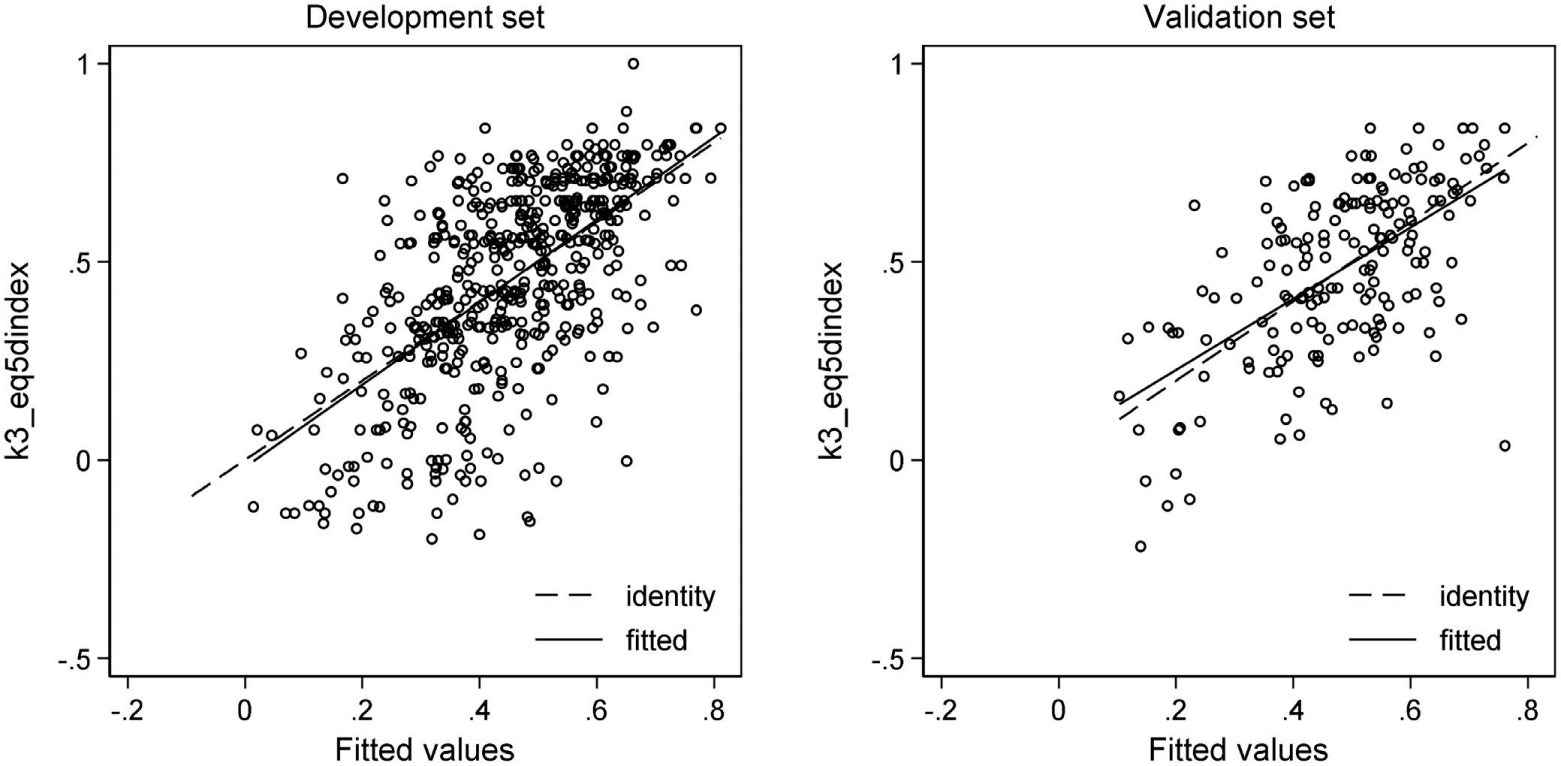
Scatter plots of the multivariable model for health‐related quality of life

ROC curves and calibration plots are shown in Figures 2, 3, 4, 5.

## 4 DISCUSSION

In this study, we developed and validated models for predicting patient‐relevant health outcomes at one year in a Norwegian cohort study of nearly 1000 patients with CWP and FM admitted for specialized rehabilitation care. The models provided poor or acceptable predictions of improvement, worsening and quality of life, and excellent predictions of working status at follow‐up.

To our knowledge, this is the largest prospective clinical prediction study that has been conducted on patients with CWP and FM. Unlike previous studies which reported prognostic factors for people with CWP and FM (Artus et al., 2017; Beneciuk et al., 2018; Ringqvist et al., 2019; de Rooij, van der Leeden, et al., 2013; Tseli et al., 2019), the current study used cross‐validated regression methods, and it externally validated estimates of predictive accuracy. The use of cross‐validated regression models and external validation increases confidence that the estimates of model performance apply to out‐of‐sample predictions (McIntosh et al., 2018; Steyerberg, 2009).

The length of the rehabilitation stay and the proportion of inpatient/outpatient rehabilitation are in line with the usual practice in specialized rehabilitation settings in Norway. Although this suggests the sample is representative of the Norwegian rehabilitation setting, the low response rate (38.7%) is still a weakness of the study. Since we have no data on non‐participants, we have few other insights into the representativeness of the study sample. The response rate in the current study is similar to that of another large cohort study conducted in Norway (response rate of 34.6%) which invited all patients, regardless of their diagnosis and health conditions, admitted to a rehabilitation centre (Moen et al., 2018).

Another limitation concerning the external validity of the study is the lack of specifications of the interventions which patients received at the rehabilitation centres. All of the centres provided physical activity/exercise, cognitive approaches, and pain management. However, we do not have more detailed information about, for example, how many sessions participants attended, or their compliance with prescribed interventions.

The proportion of participants who reported clinically relevant improvements one year after rehabilitation (8–11%) was low – much lower than the improvement which was reported in a prospective cohort study of 133 participants with CWP who received multidisciplinary treatment (48.3%) (de Rooij, van der Leeden, et al., 2013). The large difference may be explained by different outcome measures (different methods for defining improvement using the PGIC). Furthermore, sample variation, differences in the content of the multidisciplinary treatment or rehabilitation, and other contextual factors may explain this difference.

A challenge in investigating multivariable predictive models in rehabilitation is the complexity of potential predictors among rehabilitation populations (Seel et al., 2012). While demographic data have shown to be predictors for outcome after multidisciplinary treatment in patients with FM, only income status has shown to be a predictor of global perceived improvement (de Rooij, Roorda, et al., 2013). For the current study, only health predictors were included in the models. Consequently, income status was not considered as a predictor.

It is recognized that many of the potential predictors are discrete variables, and some (notably the depression, anxiety and comorbidity predictors) have just a few levels. Yet, we analysed these variables as continuous variables. Also, the effects of continuous variables were assumed to be linear and independent: non‐linear relationships between continuous predictors and outcomes were not modelled; nor were interactions between predictors. The justification for these simplifications is that in predictive models (as distinct from aetiologic models) parsimony is more important than structural correctness (Herbert, 2014).

While self‐reported physical and cognitive dimensions of health are often used as outcomes after multidisciplinary treatment or rehabilitation in patients with musculoskeletal pain, less attention has been paid to working status (Tseli et al., 2019). There was a slight decline in employment over the 12 months (an absolute decline of 6%). The design of our study does not enable us to answer questions about the effects of rehabilitation on working status. However, we found that employment before a rehabilitation stay is a strong predictor of employment at 12‐month follow‐up.

On average, outcomes slightly deteriorated over time (Klokkerud et al., 2012). Therefore, the procedure of carrying forward 6‐month outcomes for participants who did not report 12‐month outcomes may give an artificially optimistic estimate of outcomes. The degree of bias created by this procedure is, however, likely to be small.

There are some considerations to take into account if these prediction models are to be used in clinical practice. First, the objective of prediction modelling is often improved participation of stakeholders in decision‐making. Potentially our prediction models could be used as a tool for decision support in the general practitioner's office when referral to rehabilitation is being considered. However, the tool could only be used if all of the predictors were routinely available for patients with CWP and FM. Though we used lasso regression to reduce the number of predictors, the number of predictors retained in the model was still quite large, and it may not be practical to obtain data on all of the predictors. While the cost of acquiring predictor data is relatively low, patients may find that completing many questionnaires is quite tedious, and the tediousness may be unwarranted given the moderate predictive value of our models of improvement and worsening. Reasonably good predictions of quality of life at follow‐up can be made using only data on quality of life at baseline, and reasonably good predictions about work status at follow‐up can be made using only data on working status at baseline. This may be more feasible in clinical practice.

Our models do not say anything about optimum treatment. However, prediction models may eventually help clinicians select the right patient for the right form of rehabilitation.

## CONFLICTS OF INTEREST

None.

## AUTHOR CONTRIBUTIONS

V.P.M. contributed to data collection, interpretation of the data and drafted the manuscript. A.T.T. performed the statistical analysis, contributed to interpretation of the data and helped to draft the manuscript. R.H. helped planning the study, prepared for statistical analysis, contributed to interpret the data and helped to draft the manuscript. K.B.H. is the main contributor of the concept and the design of the study, contributed to interpret the data and helped to draft the manuscript. All authors discussed the results and commented on the manuscript and have approved the final manuscript.
